# Female patients with acute aortic dissection type A (AADA): A gender-selective evaluation of the intensity of the surgical treatment

**DOI:** 10.1177/02676591241303325

**Published:** 2024-11-25

**Authors:** Morsi Arar, Andreas Martens, Linda Rudolph, Heike Krueger, Victoria Arar, Erik Beckmann, Malakh Shrestha, Tim Kaufeld

**Affiliations:** 1 9177 Department of Cardiothoracic, Transplantation and Vascular Surgery, Hannover Medical School, Hannover, Germany; 2 Germany Minneapolis Heart Institute, Minneapolis, USA; 3 4352 Mayo Clinic, Rochester, MN, USA

**Keywords:** aortic, vascular, cardiac, adult, surgery, dissection, gender

## Abstract

**Objective:**

Acute aortic dissection type A (AADA) is a condition that affects both genders and requires urgent surgical intervention as non-operative treatment is often associated with a poor prognosis. Studies have proven that less aggressive surgical treatments influence the outcome for female patients in the fields of several diseases. The purpose of this study was to report and analyze our results in the selective cohort of female patients with AADA to detect differences in the treatment of this group of patients.

**Methods:**

Between January 2000 and July 2018, 141 adult female patients with acute aortic dissection (AAD) underwent repair of the aorta in our department. A total of 75 patients received a proximal arch. replacement (group A), and 66 patients received a subtotal (replacement of the brachiocephalic trunk) and/or total arch. replacement with elephant trunk or frozen elephant trunk (group B).

**Results:**

The median age in group A was 70.7 years (range 60.7–74.7 years) and in group B 66.3 years (range 55.6–71.3 years). Operation times were significantly longer in group B (total operation time: A = 286.9 min (range 225.0–341.0 min), B = 341 min (range 266.0–392 min), *p* = .003; cardiopulmonary bypass time: A = 189.0 min (range 139.0–138 min), B = 238 min (range 176.8–300.5 min), *p* < .001; circulatory arrest time: A = 27.0 min (range 21.0–37.0 min), B = 42.0 min (range 32.0–56.3 min), *p* < .001). There were no significant differences between the groups regarding 30-day mortality (A = 24% (*n* = 18), B = 19.7% (*n* = 13), *p* = .538) and rate of reoperation (A = 13.3% (*n* = 10), B = 15.2% (*n* = 10), *p* = .757) or the preoperative characteristics.

**Conclusion:**

Our study showed no significant difference in mortality rate depending on the type of operation. Based on these results, a proximal arch. replacement should be considered as the first line of operation technique if the individual situation allows. For inexperienced clinics and surgeons in particular, we recommend limited aortic repair in patients with AADA. Finally, location of the intimal tear remains the essential factor for the evaluation of the surgical treat.

## Introduction

Acute aortic dissections type A (AADA) are considered life-threatening aortic emergencies with limited outcomes. Further investigations in female patients were carried out to gain a greater understanding of the differences in the prevalence, treatment and prognosis of cardiovascular diseases according to gender.^1,2^ Furthermore, previous studies also describe existing gender-specific differences in terms of the characteristics, presenting feature and outcome in patients with aortic dissection.^3^ Nienaber et al.^4^ detected a higher rate of in-hospital complications like hypotension and tamponade resulting in a higher in-hospital mortality rate in women compared to men.^4^ Further studies have proven that less aggressive surgical treatments influence the outcome of female patients with cancer.^5^ In contrast to theses cites studies a less aggressive treatment in female patients with aortic dissection is not proven so far. The aim of this study was the evaluation of the aggressiveness of the surgical treatment of female AADA patients.

It can thus be assumed that the surgical procedure may need to be adjusted based on gender issues. Surgical procedures in patients with AADA should be evaluated to determine whether either limited or extended arch. surgery is beneficial depending on gender. The aim of this study was to investigate the outcomes and identify the risk factors associated with the surgical repair of acute thoracic aortic dissection in female patients with the goal of enhancing our understanding and informing clinical practice in this specific population.

## Material & methods

### Screening and inclusion criteria

This study was approved by the local ethics committee of the Hannover Medical School. Written informed consent from each patient was given. All data were collected retrospectively until February 2022. Patients were regularly seen in our outpatient clinic and computed tomography (CT) or magnetic resonance imaging (MRI) was performed at fixed intervals. The preoperative patient characteristics are presented in Tables 1 and 2.

**Table 1.** Patients’ characteristics: IQR (interquartile range), BMI (body mass index), PVOD (peripheral vascular occlusion disease), COPD (chronic obstructive pulmonary disease).

Characteristics	Female patients	Proximal arch replacement	Total arch/extended arch replacement	*p*-value
Female patients	*n* = 141	*n* = 75	*n* = 66	
Age at surgery (years), median (IQR)	68.5 (60.7–74.7)	70.7 (63.6–77.3)	66.3 (55.6–71.3)	**0.006**
BMI, mean ± SD	25.6 ± 4.2	25.9 ± 4.2	25.2 ± 4.2	0.388
Hypertension, n (%)	88 (62.4)	47 (62.7)	41 (62.1)	0.947
Diabetes mellitus, n (%)	14 (9.9)	10 (13.3)	4 (6.1)	0.150
PVOD, n (%)	4 (2.8)	4 (5.3)	0 (0)	0.123
COPD, n (%)	13 (9.2)	7 (9.3)	6 (9.1)	0.960
Coronary heart disease, n (%)	10 (7.1)	7 (9.3)	3 (4.5)	0.336
Hyperthyreosis, n (%)	3 (2.1)	3 (4.0)	0 (0)	0.248
Hypothyreosis, n (%)	22 (15.6)	14 (18.7)	8 (12.1)	0.285
Atrial fibrillation, n (%)	15 (10.6)	7 (9.3)	8 (12.1)	0.592
Marfan syndrome, n (%)	6 (4.3)	2 (2.7)	4 (6.1)	0.419
Bicuspid aortic valve, n (%)	6 (4.3)	3 (4.0)	3 (4.5)	1.000
Pericardial tamponade, n (%)	54 (38.3)	29 (38.7)	25 (37.9)	0.923
Preoperative intubation, n (%)	21 (14.9)	12 (16.0)	9 (13.6)	0.694
Mechanical resuscitation, n (%)	15 (10.6)	8 (10.7)	7 (10.6)	0.991
Cardiac reoperation, n (%)	4 (2.8)	4 (5.3)	0 (0)	0.123

**Table 2.** Preoperative data: CT (computed tomography), LCA (left coronary artery), RCA (right coronary artery), IQR (interquartile range).

Characteristics	Female patients	Proximal arch replacement	Total arch/extended arch replacement	*p*-value
Female patients	*n* = 141	*n* = 75	*n* = 66	
Malperfusion, n (%)	44 (31.2)	24 (32.0)	20 (30.3)	0.828
Cerebral malperfusion, n (%)	20 (14.2)	10 (13.3)	10 (15.2)	0.757
Visceral malperfusion, n (%)	14 (9.9)	7 (9.3)	7 (10.6)	0.801
Limb malperfusion, n (%)	16 (11.3)	9 (12.0)	5 (7.6)	0.185
Renal malperfusion, n (%)	12 (8.5)	6 (8.0)	6 (9.1)	0.817
Hemiparesis, n (%)	15 (10.6)	9 (12.0)	6 (9.1)	0.576
Paraparesis, n (%)	4 (2.8)	4 (5.3)	0 (0)	0.123
Seizure, n (%)	3 (2.1)	2 (2.7)	1 (1.5)	1.000
Evidence of stroke CT, n (%)	12 (8.5)	7 (9.3)	5 (7.6)	0.709
Neurological symptoms, n (%)	38 (27.0)	20 (26.7)	18 (27.3)	0.935
Dissection supra-aortic arteries, n (%)	39 (27.7)	18 (24.0)	21 (31.8)	0.300
Dissection LCA, n (%)	3 (2.1)	1 (1.3)	2 (3.0)	0.600
Dissection RCA, n (%)	18 (12.8)	8 (10.7)	10 (15.2)	0.426
Iatrogenic dissection, n (%)	5 (3.5)	3 (4.0)	2 (3.0)	1.000
Onset of pain to surgery time (h), median (IQR)	7.0 (4.8–16.5)	7.0 (4.5–14.0)	7.0 (4.8–20.3)	0.589

Inclusion criteria were the diagnosis of AADA followed by surgical treatment and patients being of female gender. All patients were treated at the Department of Cardiac, Thoracic, Transplantation and Vascular Surgery at the Hannover Medical School. The initial diagnosis was verified by CT or MRI imaging. All study patients presented a type I dissection and underwent urgent surgery. Chronic dissections as well as DeBakey II and III dissections were not included in this study. In total, 141 patients fulfilled the criteria and were included in our analysis.

Medical history, treatment including the surgical technique, and information both during and after surgery were collected at the time of the inpatient stay and the outpatient appointment.

To detect differences in the outcome and characteristics of female patients with AADA, we divided the cohort into two groups. Group A received a limited arch. repair (proximal arch. replacement) and group B received an extended arch./total arch. repair.

### Definitions

According to the DeBakey classification, acute dissection type I is defined as any dissection within the ascending aorta, the aortic arch. or the descending aorta that occurs within 14 days after symptoms.

According to Sievers et al.^6^ (TEM – Aortic Dissection Classification), occlusion or complete false lumen perfusion (stages M2 and M3 ((−), (+)) is defined as malperfusion. Coronary artery dissection was diagnosed either by intraoperative visual observation or using coronary angiography. AADAs induced during open heart surgery were defined as iatrogenic dissection. Malperfusion postoperatively detected using CT or MRI was defined as persisting malperfusion. The temporary or long-term need for kidney dialysis was defined as postoperative acute renal failure.

For the diagnosis of diabetes mellitus, hypertension or chronic obstructive pulmonary disease (COPD), a preoperatively performed medical treatment was necessary.

### Perioperative management and surgical technique

According to our standard operating procedure (SOP) for AADA, transfer to the operation room must take place promptly after diagnosis of the aortic dissection. Furthermore, we have established a rapid response team of aortic surgeons able to provide aortic repair 24/7 in cases of AADA. To avoid early cardiac decompensation, intubation was not performed until all anesthesiologic and surgical preparations were complete. This was followed by intubation and the establishment of full sternotomy extracorporeal circulation (ECC). Our team members have previously published our cannulation technique for cases of AADA.^7,8^ We preferentially chose direct aortic cannulation. Following the identification of the true lumen using transesophageal echocardiography, direct cannulation was performed. The left side of the heart was vented through the right superior pulmonary vein. The aorta was clamped if no thrombus formation was observed. Cardioplegia was administered directly into the coronary ostia. Blood cardioplegia is our favored method of myocardial protection, and we prefer a root-first procedure. The patient was cooled to a nasopharyngeal temperature of 22 °C–26 °C while the aortic/root repair was performed. Other concomitant procedures (e.g., coronary artery bypass graft, CABG) were also performed if necessary. The application of cardioplegia was performed frequently, approximately every 30 min.^8^ In all cases of AADA, either a proximal subtotal (involving the replacement of the brachiocephalic trunk) or total arch. replacement with elephant trunk (ET) or frozen elephant trunk (FET) was performed, together with hypothermic circulatory arrest (temperatures between 22°C and 26°C) and bilateral selective antegrade cerebral perfusion (SACP). The application of SACP varied when a limited arch. repair was performed.

Due to the long time frame of this study, the surgical technique regarding the choice of aortic grafts varied significantly.

### Extended arch repair

From 2000 to 2010, the FET technique was performed using a custom-made Chavan-Haverich prosthesis followed by a prefabricated Chavan-Haverich hybrid graft^9,10^ (Curative GmbH, Dresden, Germany). Furthermore, we used the JOTEC E-vita hybrid graft from 2005 to 2010.^11^ Supra-aortic artery attachments were performed using the island (en bloc) technique until 2010. After the island technique, we switched to the four-branched FET graft (FET Vascutek Terumo, Terumo®, Glasgow, UK). In 2007, we changed our approach from a straight graft using the island technique to the branched Sienna™ graft (Terumo®, Glasgow, UK), also for total or hemi-arch replacements. The widespread use of branched aortic arch. prostheses has led to important technical developments. As a result of these changes, arch. replacement was performed after completion of cardiac and proximal aortic repair. Head vessels were anastomosed to the corresponding side branches of the graft at the end of the procedure.^12^ An extended arch repair is characterized by a distal anastomosis located in zones one and two of the aortic arch.

### Proximal arch repair

An isolated replacement of the proximal aortic arch. was performed using different straight Dacron grafts. The proximal arch. repair was defined as a distal anastomosis located in zone 0. All cases a proximal arch. replacement were performed under circulatory arrest.

### Statistical analysis

SPSS Statistics 27 software (IBM Corp. Released 2020; IBM SPSS Statistics for Windows, Version 27.0; Armonk, NY: IBM Corp.) was used for the data analysis. A normal distribution of variables was calculated using the Kolmogorov–Smirnov test. Categorical variables were given as absolute numbers (n) and proportions. Normally distributed continuous variables were given as mean ± standard deviation, while continuous variables without normal distribution were given as the median and interquartile range (IQR). Fisher’s exact test was used to detect differences in the categorical variables. Differences in the continuous variables were tested using the Mann-Whitney *U* test. Kaplan–Meier analysis and log rank were used for the evaluation of survival, and the log rank test was used to test for differences. We did not correct for multiple testing. A univariable analysis was performed to test for any association between the variables and in-hospital mortality.

## Results

The study included 141 female patients with acute aortic dissection (AAD) DeBakey type I who underwent surgery at our department between January 2000 and July 2018. Median age in group A was 70.7 years (range 60.7–74.7 years) and in group B 66.3 years (range 55.6–71.3 years).

All intraoperative data are listed in Table 3. Patients in group A (*n* = 75) received a replacement of the ascending aorta and the proximal aortic arch. Patients in group B (*n* = 66) underwent extended aortic arch. repair including a subtotal arch. replacement or total arch. replacement as well as ET or FET prosthesis. As concomitant procedures, aortic valve surgery (aortic valve replacement, root replacement with a composite graft and aortic root reimplantation) or aortic valve reconstruction (David procedure) were performed in both groups. The number of concomitant procedures performed was not significantly different between the groups.

**Table 3.** Intraoperative data: SD (standard deviation), IQR (interquartile range), min (minute), HCA (hypothermic circulatory arrest time), CABG (coronary artery bypass graft), SACP (selective antegrade cerebral perfusion time).

Characteristics	Female patients	Proximal arch replacement	Total arch/extended arch replacement	*p*-value
Female patients	*n* = 141	*n* = 75	*n* = 66	
Total operation time (min), median (IQR)	307.0 (248.5– 370.0)	286.0 (225.0–341.0)	341.0 (266.0–392.0)	**0.003**
Cardiopulmonary bypass time (min), median (IQR)	203.0 (165.0–259.5)	189.0 (139.0–137.0)	238.0 (176.8–300.5)	**<0.001**
Aortic cross-clamp time (min), mean ± SD	118.4 ± 46.0	111.7 ± 39.6	126.1 ± 51.5	0.064
HCA (hypothermic circulatory arrest) time (min), median (IQR)	33.0 (24.0–46.0)	27.0 (21.0–37.0)	42.0 (32.0–56.3)	**<0.001**
SACP (selective antegrade cerebral perfusion) time (min), median (IQR)	28.0 (17.0–64.5)	22.0 (13.0–29.0)	64.5 (28.8–90.3)	**<0.001**
Minimal core temperature (°C), median (IQR)	24.7 (22.3–26.0)	25.0 (22.9–26.8)	24.2 (22.0–25.4)	0.064
Erythrocyte concentrates, median (IQR)	6.0 (4.0–10.0)	6.0 (3.0–9.0)	7.0 (4.0–10.0)	0.054
Fresh frozen plasma, median (IQR)	6.0 (4.0–8.0)	6.0 (4.0–8.0)	6.0 (4.0–9.0)	0.114
Platelet concentrates, median (IQR)	2.0 (2.0–3.0)	2.0 (2.0–3.0)	2.0 (2.0–4.0)	**0.033**
Proximal arch replacement, n (%)	75 (53.2)	75 (100.0)	0 (0)	**-**
Subtotal arch replacement, n (%)	14 (9.9)	0 (0)	14 (21.2)	**<0.001**
Total arch replacement, n (%)	13 (9.2)	0 (0)	13 (19.7)	**<0.001**
Total arch replacement elephant trunk, n (%)	12 (8.5)	0 (0)	12 (18.2)	**<0.001**
Total arch replacement frozen elephant trunk, n (%)	27 (19.1)	0 (0)	27 (40.9)	**<0.001**
Bio glue, n (%)	55 (39.0)	30 (40.0)	25 (37.9)	0.797
Aortic valve replacement bio, n (%)	24 (17.0)	15 (20.0)	9 (13.6)	0.316
Aortic valve replacement mech, n (%)	15 (10.6)	7 (9.3)	8 (12.1)	0.592
Root involvement, n (%)	79 (56.0)	41 (54.7)	38 (57.6)	0.728
Bentall, n (%)	38 (27.0)	21 (28.0)	17 (25.8)	0.765
David, n (%)	27 (19.1)	13 (17.3)	14 (21.2)	0.559
Yacoub, n (%)	9 (6.4)	5 (6.7)	4 (6.1)	1.000
CABG, n (%)	29 (20.6)	14 (18.7)	15 (22.7)	0.552
ECMO postoperative, n (%)	8 (5.7)	3 (4.0)	5 (7.6)	0.474
Death on the operating table, n (%)	4 (2.8)	2 (2.7)	2 (3.0)	1.000

Due to the extended surgical procedure, total operation time (A = 286.9 min (range 225.0–341.0 min), B = 341 min (range 266.0–392 min), *p* = .003), cardiopulmonary bypass time (CPB) (A = 189.0 min (range 139.0–138 min), B = 238 min (range 176.8–300.5 min), *p* < .001) and circulatory arrest time (A = 27.0 min (range 21.0–37.0 min), B = 42.0 min (range 32.0–56.3 min), *p* < .001) were significantly longer in group B. Mean nasopharyngeal temperature at the induction of circulatory arrest was slightly but not significantly lower in group A (A = 24.2°C (range 22.0 °C–25.4 °C), B = 25.0°C (range 22.9 °C–26.8 °C), *p* = .064). Corresponding to the extent of surgery, median SACP time was significantly increased in group B (A = 22 min (range 13.0–29.0 min), B = 64.5 min (range 28.8–90.3 min), *p* < .001).

The necessity for extracorporeal membrane oxygenation (ECMO) support (A = 4% (*n* = 3), B = 7.6% (*n* = 5), *p* = .474) and death on the operating table (A = 2.7% (*n* = 2), B = 3% (*n* = 2), *p* = 1.000) did not differ significantly between the groups (Table 4).

**Table 4.** Postoperative data: SD (standard deviation), IQR (interquartile range), min (minute), CCT (cranial computed tomography).

Characteristics	Female patients	Proximal arch replacement	Total arch/extended arch replacement	*p*-value
Female patients	*n* = 141	*n* = 75	*n* = 66	
Survival time (days), median (IQR)	1534 (63.5–2988.5)	1613.0 (32.0–3393.0)	1523.0 (163.3–2752.0)	0.724
Ventilation time (h), median (IQR)	45.0 (19.5–125.5)	36.0 (18.0–120.0)	54.0 (24.8–131.8)	0.333
Intensive care unit (days), median (IQR)	4.0 (2.0–7.5)	4.0 (2.0–7.0)	5.0 (2.0–8.0)	0.168
Rethoracotomy, n (%)	22 (15.6)	9 (12.0)	13 (19.7)	0.209
Dialysis, n (%)	20 (14.2)	9 (12.0)	11 (16.7)	0.428
30-day mortality, n (%)	31 (22.0)	18 (24.0)	13 (19.7)	0.538
CCT stroke, n (%)	38 (27.0)	16 (21.3)	22 (33.3)	0.109
New-onset stroke, n (%)	14 (9.9)	6 (8.0)	8 (12.1)	0.414
Persisting cerebral malperfusion, n (%)	7 (5.0)	1 (1.3)	6 (9.1)	0.051
Persisting limb malperfusion, n (%)	5 (3.5)	4 (5.3)	1 (1.5)	0.371
Persisting visceral malperfusion, n (%)	3 (2.1)	2 (2.7)	1 (1.5)	1.000
Persisting renal malperfusion, n (%)	8 (5.7)	4 (5.3)	4 (6.1)	1.000

### Postoperative data

Overall, the postoperative results of both cohorts were comparable. In particular, median survival time (A = 1613 d (range 32.0–3393.0 d), B = 1523.0 d (range 163.3–2752.0 d), *p* = .724) and 30-day mortality (A = 24% (*n* = 18), B = 19.7% (*n* = 13), *p* = .538) showed no significant differences. Correlating with the extent of the surgical procedure, median ventilation time was prolonged in group B (A = 36.0 h (range 18.0–120.0 h), B = 54.0 h (range 24.8–131.8 h), *p* = .333). The rate of postoperative acute renal failure was 19.7% (*n* = 13) in group B and 12% (*n* = 9) in group A.

Neurological complications such as stroke (A = 21.3% (*n* = 16), B = 33.3% (*n* = 22), *p* = .109) were not significantly increased in group B. The proportion of new-onset stroke in group B was also slightly but not significantly higher (A = 8.0% (*n* = 6), B = 12.1% (*n* = 8), *p* = .414). Although persisting malperfusion was almost equally distributed in both cohorts, it nevertheless represents an exception to this. Despite the extended arch. repair, postoperative persisting malperfusion occurred more frequently in group B (A = 1.3% (*n* = 1), B = 9.1% (*n* = 6), *p* = .051).

### Follow-up and long-term outcomes

The operative reports of all 141 patients were evaluated and follow-up was 100% completed. Overall, no significant differences were detected regarding secondary aortic operations. During follow-up, reoperation was indicated in 14.2% (*n* = 20) of all study patients (A = 13.3% (*n* = 10), B = 15.2% (*n* = 10), *p* = .757). Despite the extended arch. operation, the requirement for reoperation in the downstream aorta was comparable in both groups (A = 8.0% (*n* = 6), B = 10.6% (*n* = 7), *p* = .594). Two of the study patients in group B were treated with an aortic fenestration. Furthermore, seven patients received a secondary endovascular treatment (thoracic endovascular aortic repair (TEVAR): A = 1.3% (*n* = 1), B = 4.5% (*n* = 3), *p* = .340; endovascular aneurysm repair (EVAR): A = 1.3% (*n* = 1), B = 3.0% (*n* = 2), *p* = .600). Follow-up data are listed in Table 5.

**Table 5.** Follow-up data: TAA (thoracoabdominal repair), TEVAR (thoracic endovascular aortic repair), EVAR (endovascular aneurysm repair).

Characteristics	Female	Proximal arch replacement	Total arch/extended arch replacement	*p*-value
Female patients	*n* = 141	*n* = 75	*n* = 66	
Secondary aortic operation, n (%)	20 (14.2)	10 (13.3)	10 (15.2)	0.757
Reoperation identical area, n (%)	7 (5.0)	4 (5.3)	3 (4.5)	1.000
Reoperation downstream aorta, n (%)	13 (9.2)	6 (8.0)	7 (10.6)	0.594
TAA repair, n (%)	4 (2.8)	2 (2.7)	2 (3.0)	1.000
Y prosthesis, n (%)	1 (0.7)	0 (0.0)	1 (1.5)	0.468
Descending repair, n (%)	4 (2.8)	2 (2.7)	2 (3.0)	1.000
Hybrid, n (%)	2 (1.4)	2 (2.7)	0 (0.0)	0.498
TEVAR, n (%)	4 (2.8)	1 (1.3)	3 (4.5)	0.340
EVAR, n (%)	3 (2.1)	1 (1.3)	2 (3.0)	0.600
Aortic fenestration	2 (1.4)	0 (0.0)	2 (3.0)	0.217

### Survival analysis

**Figure 1.**
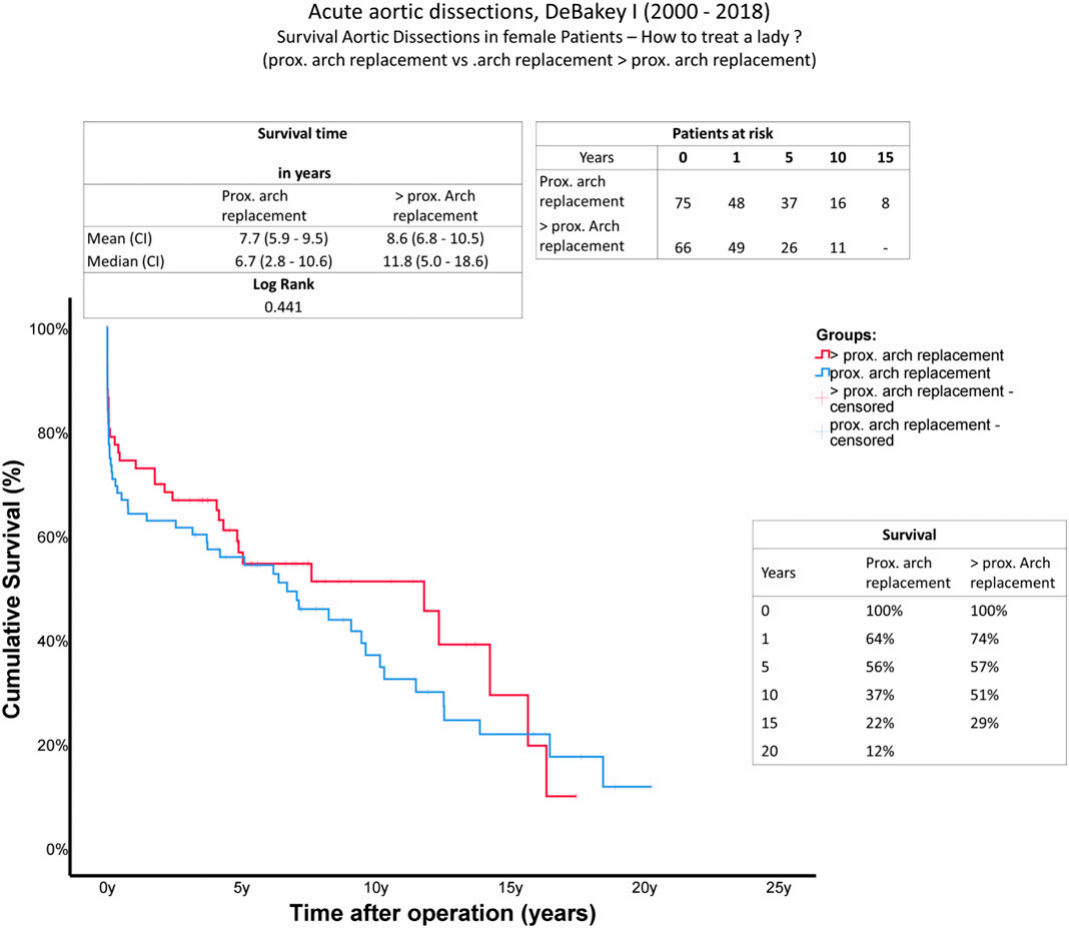
Survival: Kaplan-Meier curves showing survival of female patients after surgical treatment of AADA. The *x*-axis denotes the time after operation.

A comparison of long-term survival using Kaplan-Meier curves can be found in Figure 1, showing data from all 141 patients, including patients who died during surgery (*n* = 4). Mean survival (A = 7.7 years (range 5.9–9.5 years), B = 8.6 years (range 6.8–10.5 years)) and median survival (A = 6.7 years (range 2.8–10.6 years); B = 11.8 years (range 5.0–18.6 years)) were reduced in group A (log rank 0.441). The 10-year survival rate was higher in group B (A = 37%, B = 51%).

## Discussion

AAD is often a fatal condition, especially for female patients. Huckaby et al. demonstrated that according to the International Registry of Acute Aortic Dissection, female patients present later and tend towards higher in-hospital mortality overall.^3^ Furthermore, the cohort of older female patients presenting with dissection are also more likely to present with intramural hematoma, periaortic hematoma, or complete or partial false lumen thrombosis, and more commonly had hypotension or coma admissions than men.^3^ Taking into account that the treatment of diverse pathologies varies due to patients’ gender,^1,4,5,13^ the surgical repair of AADA, especially for female patients, requires further evaluation.

According to our data, the main factor in the decision concerning surgical extent is age. Younger female patients are more likely to benefit from an extended arch. repair. The findings of Qin et al.^14^ support the thesis that limited arch. repair in cases of AADA is justified in elderly patients. The age of the limited arch. repair group (70.7 years) was identical to our data. In our study, age was the only relevant preoperative factor that differed significantly between the groups (A = 75 years, B = 66 years, *p* = .006). Age remains a relevant factor when evaluating the type of surgical treatment required. Existing studies emphasize the option of less invasive surgery or a more conservative strategy in cases of AADA in elderly patients.^15^

In our cohort, the preoperative conditions of patients and the chosen surgical procedure were equally distributed. Accordingly, any specific adjustments to the surgical procedure for the patient’s conditions apart from the age factor was not discerned.

It is reasonable that procedural times were significantly longer in the extended arch. repair group. In the German Registry for Acute Aortic Dissection Type A, Boening et al.^16^ describe how longer operating and bypass times were independent risk factors for neurological dysfunction after AADA.

Based on our data, ventilation time and duration of intensive care unit treatment also corresponded to the extent of the surgical treatment performed.

The frequency of postoperative complications like rethoracotomies and renal failure was also positively, but not significantly, associated with the extent of complex surgery.

The decision regarding the extent of the surgical procedure is also associated with the existence of preoperative malperfusion and expectations relating to persisting malperfusion after surgery. Persisting malperfusion after surgery on its own is an independent risk factor for early death.^17^ Neither the number of patients with persisting visceral malperfusion (A = 2.7%, B = 1.5%, *p* = 1.0), nor persisting limb malperfusion (A = 5.3%, B = 1.5%, *p* = 1.0) were significantly reduced in the group with total arch. repair or even FET. A higher incidence of preoperative cerebral malperfusion also leads to an increased number of female patients with persisting postoperative cerebral malperfusion, despite adequate surgical treatment of the supra-aortic artery during the total arch. replacement.

However, we found that limited aortic arch. repair is a reasonable option in female patients, even in a female cohort of advanced age. Particularly in cases of a perioperative stroke, tamponade or complex anatomy, a proximal arch. replacement seems to be an adequate alternative. Regarding existing gender differences, Li et al.^18^ found shortened long-term survival in women after AADA in general. Median survival time was increased in the extended arch. repair group (A = 6.7 years (range 2.8–10.6 years), B = 11.8 years (range 5.0–18.6 years), log rank = 0.441), which should be considered against the background of the higher age of group A. Long-term mortality may be slightly elevated in this “proximal arch.” group due to the greater age of this cohort at the time of dissection. Medium-term results after 5 years seem to be almost equal between both groups. According to the Kaplan-Meier curves, a clear benefit of a total arch. repair was observed 10 years after surgery.

It can be assumed that one intention of total arch. repair is to avoid operations in the downstream aorta in the further course of treatment. Despite this assumption, no significant differences regarding second-stage therapy were detected. In conclusion, our data indicate that limited initial aortic repair in AADA cases is certainly warranted in female patients, given that extended repair does not correlate with a reduced rate of re-operation. In particular, inexperienced centers and surgeons should evaluate this option to achieve the first aim of the surgical AADA treatment: survival. Furthermore, limited arch. repair also remains an adequate option for an elderly and/or more compromised female cohort. Nevertheless, the location of the intimal entry tear represents the most important factor for the evaluation of arch. repair extent.

## Limitations

Because this is a retrospective study, it carries the potential risks and biases linked to studies of this nature. Furthermore, the individual decisions regarding surgical procedures were based on the surgeon’s experience. Between the years 2010 and 2018, a total of 19 surgeons performed the operative treatment of these patients. Surgical skill levels may have varied in this cohort. Multicenter studies are needed to suggest whether limited aortic repair may be warranted in female patients with AADA. In addition, a relevant number of patients who died before admission can be expected.

## Conclusion

AADA is one of the most dangerous and fatal cardiovascular emergencies in any gender, but with a more aggressive and complicated course in female patients, it has a proven higher degree of in-hospital mortality in female patients. Previous studies assumed a greater age was associated with more life-threatening preoperative conditions in female patients with AADA compared to men.

The focus of this study was to evaluate the consequences of the surgical technique (limited aortic arch. repair vs extended arch. repair), specifically in the more fragile cohort of female patients with AADA.

In summary, female patients with AADA neither received a less invasive treatment (proximal arch. replacement) nor was an extended arch. replacement preferred. The factor age was the deciding factor in the selection of the surgical procedure.

This study demonstrates that in appropriately selected patients with further risk factors (age, morbidities) a less invasive treatment in terms of early and long-term outcomes.

An extended arch. repair is associated with longer operative time, CPB time and circulatory arrest time. According to these results, indication of an extended arch. repair should be evaluated carefully. The presence of connective tissue diseases, an intimal tear in the aortic arch., or severe malperfusion in the downstream aorta are dependable indicators for considering a total arch. replacement or a frozen elephant trunk procedure when suitable.

## Appendix

### Abbreviations

AAD: Acute aortic dissection
AADA: Acute aortic dissection type A
BMI: Body mass index
CABG: Coronary artery bypass graft
COPD: Chronic obstructive pulmonary disease
CCT: Cranial computed tomography
CT: Computed tomography
d: Days
ECMO: Extracorporeal membrane oxygenation
ET: Elephant trunk
ECC: Extracorporeal circulation
EVAR: Endovascular aneurysm repair
FET: Frozen elephant trunk
HCA: Hypothermic circulatory arrest
IQR: Interquartile range
LCA: Left coronary artery
min: Minute
MRI: Magnetic resonance imaging
n: Number
PVOD: Peripheral vascular occlusion disease
RCA: Right coronary artery
SACP: Selective antegrade cerebral perfusion
SD: Standard deviation
SOP: Standard operating procedure
TAA: Thoracoabdominal repair
TEVAR: Thoracic endovascular aortic repair
y: Years
